# Atlanta Medico-Chirurgical Association

**Published:** 1878-02-20

**Authors:** 


### ATLANTA MEDICO-CHIRURGICAL ASSOCIATION.

The above association met on Friday night, the 8th instant, Dr. G. G. Crawford in the chair.

On the call for report of cases, Dr. T. S. Powell stated that he had read a very interesting paper by Dr. L. P. Yandell, Jr., Professor of Clinical Medicine and Therapeutics in the University of Louisville, on "Malaria and Struma; their Relation to the Etiology of Skin Diseases," in which paper the distinguished author contends that "Malaria is the chief source of acute skin diseases, and that scrofula is the chief source of chronic skin diseases." Dr. P. stated that while he was not prepared to endorse fully all of the results of Dr. Yandell's clinical experience upon this subject, it afforded him pleasure to say that he had for twenty years treated many forms of skin disease with the idea that they were caused by malaria, and had observed the fact that they were more amenable to quinine and iron than to other remedies. Hence he was disposed to regard Dr. Yandell's theory as highly probable. He referred to several cases of recent date in his practice as confirming the malarial origin of skin affections. The first was a child nine years old, who had been declining in health for a year or more, and had been variously treated for dropsy, rheumatism, etc. She became sallow, feeble, and finally anæmic. During this time she was a dancing scholar, and danced frequently until exhausted. After this there appeared a hemorrhage from the bladder and an eruption upon the skin. The hemorrhage has recurred at intervals, and the eruption is more plainly marked immediately after exercising. The question now arises, did the general bad health and anæmic condition result from latent malaria, as well, also, the eruption and hemorrhage—the undue exercise and over-heating being the exciting cause which developed the symptoms?

Dr. P. stated that he could cite other cases tending to confirm Dr. Yandell's theory, but only desired at present to bring to the notice of the Association this new theory relating to the influence of malaria and struma in the production of skin diseases. He concluded by asking the views of members as to the possibility of malaria producing acute skin diseases, as it does affections of other organs and tissues. If Dr. Yandell's theory is true, it is highly important that it be known to the profession, as calculated to facilitate greatly the investigation and diagnosis of these affections, and to open the way for more direct and efficient results in their treatment.

Dr. A. R. Alley replied to Dr. Powell that he had not seen Dr. Yandell's paper, but thought the theory of the malarious origin of skin diseases was strange and untenable, because, in malarial districts, where the generation of this toxical agent occurs, we have an array of symptoms well marked and decided, characteristic of its effects, to-wit: chills and fever and their sequence. He had not noticed, when practicing in malarial belts, skin diseases produced by this poison. He held that all skin affections were the result of a hereditary diathesis, either strumous or syphilitic, or some peculiar, unexplained condition of the system. Could we reasonably say that the eruption of measles and scarlatina, known to be the result of a peculiar and specific poison, were produced by malaria?

Dr. Powell replied that Dr. Yandell did not hold that miasm was the sole cause of skin diseases, but that he regarded it a chief source of acute skin diseases. He admitted that many had special causes, and that a variety of causes might produce eruptive affections. Some of these were perhaps merely exciting causes, malaria being the remote cause. The question, then, is, can malaria be the chief cause of acute skin diseases?

Dr. J. J. Knott did not regard the cases reported by Dr. P. as necessarily indicating the miasmatic source of skin affections. He thought that more had been put upon the shoulders of malaria than was justly due. He believed that derangement of the alimentary canal was the most frequent cause of eruptive diseases. Remedies directed to the improvement of the secretions, and the general health, would be likely to benefit eruptive diseases. Hence the good resulting from iron and quinine in the cases mentioned.

Dr. R. C. Word stated that he had not read the views of Dr. Yandell, but was satisfied that malaria was the cause of many diseased conditions which we were not accustomed to attribnte to it. In this climate, particularly, it is potent for evil, and the phases which it assumed were numerous. The theory of Dr Yandell was a novel one, but by no means improbable. He had recently treated a case of seeming paralysis in a child three years old. The child was scarcely able to stand or walk—the eyes were at times crossed with other spasmodic symptoms, and the paralytic condition was more strongly marked on one side of the body. Supposing that these symptoms were due to reflex irritation from worms, he commenced the treatment with vermifuges, but no worms were discharged. Noticed that there was a somewhat feverish and restless condition in the afternoon and at night, which occurred regularly. Learned that about four weeks previous an eruption had existed, supposed to be chicken-pox, but had disappeared a short while before the paralysis was developed. On learning these facts, he commenced the use of quinine, from which time the improvement was marked and rapid, the febrile exacerbations ceased, and the child soon regained the use of its limbs.

That malaria was the prime, perhaps the sole agent in producing the varied and peculiar symptoms of this case, there can be little doubt.

Dr. Roach stated that the theory of the malarious origin of skin affections, was new to him. He thought scrofula and syphilis were productive of skin diseases. In the case mentioned by Dr. Word, he did not think the eruption was due to malaria.

Dr. C. L. Olmstead said his experience was limited, and he could not speak from observation, but if the blood was affected by malaria, as all admit, and if the skin is nourished and affected by the blood, as other tissues are, he could see no reason why it might not also become diseased from malaria, as other organs are.

